# Heaven It's My Wife! Male Canaries Conceal Extra-Pair Courtships but Increase Aggressions When Their Mate Watches

**DOI:** 10.1371/journal.pone.0022686

**Published:** 2011-08-09

**Authors:** Davy Ung, Mathieu Amy, Gérard Leboucher

**Affiliations:** Laboratoire d'Ethologie et Cognition Comparées, EA 3456, Université Paris Ouest Nanterre La Défense, Paris, France; Université Pierre et Marie Curie, France

## Abstract

Many animals live in a communication network, an environment where individuals can obtain information about competitors or potential mates by observing interactions between conspecifics. In such an environment, interactants might benefit by changing their signalling behaviour in the presence of an audience. This audience effect seems widespread among species, has been observed during various types of interaction (e.g. intra-sexual vs. inter-sexual interaction) and varies according to the social context (e.g. gender, hierarchical or mating status of the audience). However, the way individuals might adapt their signalling behaviour to a combination of these factors remains poorly understood. To address this question, we studied how the presence of an audience affects the behaviour of male domestic canaries *Serinus canaria* during two types of interactions: (i) an extra-pair interaction and (ii) a male-male competition for food. Males were observed under three conditions: (a) in the absence of audience, (b) in the presence of their mate or (c) of a familiar female. Our results show that male domestic canaries minutely adapt their courting and agonistic behaviours to a combination of: (i) the type of interaction (extra-pair interaction/male-male competition), (ii) the social context (mate, familiar female or nobody in audience) and (iii) the behaviours of both the audience and the interactant. These results highlight the ability of animals to subtly adapt their behaviour to the social environment. This also raises questions about the cognitive foundations and evolution of these processes especially considering that canaries are known neither for having high cognitive abilities nor for being a typical example for the social intelligence hypothesis.

## Introduction

Many animals live in a communication network: an environment where the distance between individuals is shorter than the range of communication signals [1]. In such an environment, clues and signals resulting from interactions between individuals are available to a third party. Therefore, individuals can extract relative information about the motivation, status or quality of conspecifics by observing their interactions; i.e. they eavesdrop [1], [2]. Males of various species have been found to eavesdrop on male-male interactions and use the information gathered in subsequent encounters (e.g. nightingale, *Luscinia megarhynchos* [3], [4]; fighting fish, *Betta splendens* [5]; great tit, *Parus major* [6]; domestic canary, *Serinus canaria* [7], [8]). Females also seem to evaluate potential sexual partners by eavesdropping both during the initial stages of mate choice (fighting fish [9]; Japanese quail, *Coturnix japonica* [10], [11]; domestic canary [12], [13]) and during extrapair attempts (great tit: [14]; black-capped chickadee, *Poecile atricapilla* [15]). Thus, the information obtained by eavesdropping can modify the fitness of individuals by influencing the agonistic behaviours an animal undergoes or its reproductive success.

Because of eavesdropping, controlling the information available to conspecifics should be as important as obtaining information. Such selection pressures on the signalling behaviour may account for audience effects. Audience effects have been defined as the changes in the signalling behaviour of an individual engaged in an interaction because of the mere presence of an audience; i.e. a conspecific witnessing the interaction [16], [17], [18]. Audience effects can conceal (e.g. [16]), highlight (e.g. [19]) or reduce the reliability (e.g. [20]) of the information available to a third party. For instance, during male-male competitions, male guppies (*Poecilia reticulate*) [21] and field crickets (*Gryllus bimaculatus*) [22] become more aggressive in the presence of a female while male fighting fishes decrease aggressive displays directed solely to males but increase conspicuous displays used with both males and females [16]. Male fighting fishes also become more aggressive in the presence of a male audience compared to the presence of a female [23] and at last, the presentation of an audience before the beginning of an interaction could lead to priming effect of agonistic behaviours [24]. During parent-offspring interactions, male vervet monkey (*Chlorocebus pygerythrus*) altered their affiliative and agonistic behaviours toward the offspring in the presence of the infant's mother [25]. Few studies investigated such audience effects during male-female interactions even if they turn out to be essential for the reproductive success of individuals. In chimpanzees (*Pan troglodytes*), calls that females utter during copulation are modulated by the proximity and the hierarchical status of female audiences [26] while subordinate rams mount and ejaculate less when viewed by dominant individuals [27]. During pair interactions, male zebra finches (*Taeniopygia guttata*) respond more to partner's voice if a pair is in audience [28] while males rock sparrow (*Petronia petronia*) increase their rate of courtship displays towards their mate when simulated courtship interactions take place in the vicinity of their nest [29]. At last, during extra-pair interactions, male budgerigars (*Melopsittacus undulates*) decrease their extra-par courtships in the presence of their partner [30]. However, in this study, individuals could communicate vocally even when the partner was not supposed to be in audience.

Thus, audience effects have been observed during various types of interaction and are sensitive to social context. However, little is known about the way individuals might adapt their signalling behaviour to a combination of these factors. In the present study, we investigate if male canaries could adjust their audience effect to a combination of both the type of interaction (an extra-pair interaction or a male-male competition) and the social context (mate or familiar female in audience).

We used domestic canaries *Serinus canaria* because they represent a good model to address this question. Canaries are socially monogamous [31], they can discriminate their mate from a familiar individual [32] and both wild and domestic canaries have been observed engaging in extra-pair copulations [33]. Furthermore, female canaries eavesdrop on vocal and physical contests between males and use the obtained information to direct their sexual behaviours [12], [13]. Although audience effects have never been explored in this species, it is likely that, a least, male-male interactions are modified by the presence of females.

The presence of the mate as an audience could impose specific pressures on interacting males according to the type of interaction. In an extra-pair context, as suggested by Baltz & Clark [30], females could evaluate the quality of their mate using their extra-pair behaviours as an indicator of future paternal investment. Thus, we expected that, during an extra-pair interaction, males should exhibit an audience effect on courting behaviours: they should court less in the presence of their mate than in the presence of a familiar female or without an audiene. Contrary to male-female interactions which are mostly affiliative, male-male competitions are essentially agonistic. Furthermore, the presence of their mate could induce mate guarding in males [34]. At last, during a male-male competition, females who see their mate losing a contest engage more in extra-pair copulations [15]. Thus, during a male-male competition, males should exhibit an audience effect on agonistic behaviours: they shoud be more aggressive in the presence of their mate than in the presence of a familiar female or without an audience.

To test these predictions, we observed male domestic canaries during two types of interactions: (i) with a sexually receptive female and (ii) with another male during a competition for food. For both these experiments, subjects were observed under three successive conditions: (a) without an audience, (b) in the presence of their mate and (c) in the presence of a familiar female.

## Methods

### Animals and breeding conditions

We randomly paired 21 male and 21 unfamiliar and unrelated female adult domestic canaries (*Serinus canaria*). All individuals were born and bred in our laboratory, had reproductive experience and were naive to testing procedures. Each pair was housed in a cage (59×50×50 cm) and was provided *ad libitum* water, mash, seeds and cotton fibers as nesting materials. To avoid familiarity bias, we kept cages in the same ‘breeding room’so that all animals would be familiar with each other. After seven days of cohabitation, we raised the light∶dark cycle to 15∶9 h to stimulate reproductive behaviours [35]. Animals performed a first reproductive cycle that enhanced pair-bonding. During this period, we replaced the layed eggs by plastic decoys so that none of the pairs would reproduce. This precaution avoided to induce a bias since the reproductive success experienced during a cycle might influence both sexual preferences [32] and the extra-pair paternity rate [36] during the following reproductive period. One week after the last egg was layed, we removed the nests. This initiated the second reproductive cycle during which we performed the experiments.

### Ethics statement

During the first reproductive cycle, we removed eggs the day of laying, when the nervous system of the embryo is not developed yet. To avoid injuries during the interactions, we monitored experiments at all times and decided to stop the test, separate the birds and discard the data when one bird pecked another bird more than 10 times. In practice, this situation never occurred because the interaction cage was large enough to allow the birds to escape from another. In addition, our protocol required to handle the birds to put them back in aviaries after the experiments. We checked animals at this moment and never observed visible injury. For the competition experiment, males were food-deprived for two hours. As a precaution, we never performed this experiment before noon so that animals had all the morning to feed before the food-deprivation. Experimental authorization was delivered by the French Ministry for Agriculture and Fisheries (Gérard Leboucher, authorization no. 92-230).

### Experiment 1: effect of a mate/familiar audience on the extra-pair behaviour of males

#### Subjects

We used the 21 males (as subjects) and 21 females (as audiences) from the previously formed pairs. Experiments took place during females' period of sexual responsiveness to stimulate males' motivation to interact.

#### Experimental design

**Figure 1:**
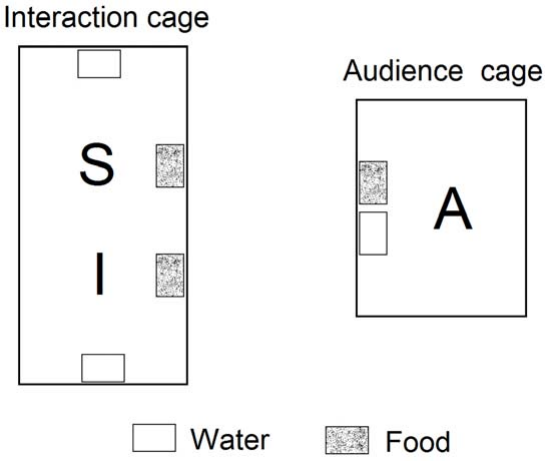
Experimental setup used in experiments 1 and 2. In both experiment, subjects (S) were placed in a cage where they could interact with an individual (I) in front of another cage where an audience (A) was present or not. In experiment 1 subjects could interact with a receptive female while in experiment 2, subjects were in a competition for food with another male. In both experiment, there were three conditions: a ‘no audience’ condition, b ‘familiar audience’ condition and c ‘mate audience’ condition. The order of conditions was balanced.

We placed the tested male in a cage (55×28×33 cm) with a sexually receptive ‘interacting female’; a grid separated individuals preventing physical interactions such as aggressions or copulations. After a 30 minutes familiarization period, we removed the grid and animals could interact during three 10 minutes successive phases. At the beginning of each phase, we placed an ‘audience cage’ (40×24×30 cm) in front of the interaction cage allowing individuals to see and hear each other. According to the phase, the audience cage: (i) was empty, (ii) contained a familiar female, or (iii) the mate of the subject (Fig. 1). The order of phases was balanced between experiments. We assessed males' responses by counting songs, trills, mild arousal and attack calls [37], copulation attempts, initiated threats and attacks and foraging behaviours. We also measured the behaviours of both the interacting female and the audiences. To avoid a possible experimenter bias, the observer did not know the identity of the audiences (mate or familiar) until data were analysed.

#### Data analysis

For all individuals, we performed a principal component analysis (PCA) that provided synthetic measures of agonistic and affiliative behaviours. After normalization of data, we performed two linear mixed models (LMM). LMM n°1 had males' affiliative behaviours as dependant variable, (ii) the experimental condition and behaviours of both interacting and audience females as fixed effects and (iii) the identity of subjects as a random effect to cope with the repeated measures. LMM n°2 was similar but performed on males' agonistic behaviour. We ended carrying out *post hoc* analysis using ANOVAS for repeated measures followed by Student-Newman-Keuls tests (SNK). We used R© 2.9.0 (The R Foundation for Statistical Computing, Vienna, Austria) for all statistical analysis.

#### Results

The PCA we performed on males' behaviours had two main axes. The first axis explained 29.41% of variance (eigenvalue: 2.35) and regrouped agonistic behaviours (eigenvectors: attack calls: 0.81; initiated threats: 0.93; initiated attacks: 0.75). The second axis explained 20.27% of the variance (eigenvalue: 1.62) and regrouped affiliative behaviours (eigenvectors: songs: 0.81; copulation attempts: 0.75). Both these synthetic variables followed a Johnson's Su distribution (affiliative behaviours: γ = −0.24; δ = 0.73; θ = −0.31; σ = 0.13; agonistic behaviours: γ = −0.39; δ = 0.54; θ = −0.57; σ = 0.32) what allowed us normalizing them using Johnson's transforms [38] (Shapiro-Wilk tests: affiliative behaviours: W = 0.98; N = 21; p = 0.86; agonistic behaviours: W = 0.97; N = 21; *p* = 0.17).

**Figure 2:**
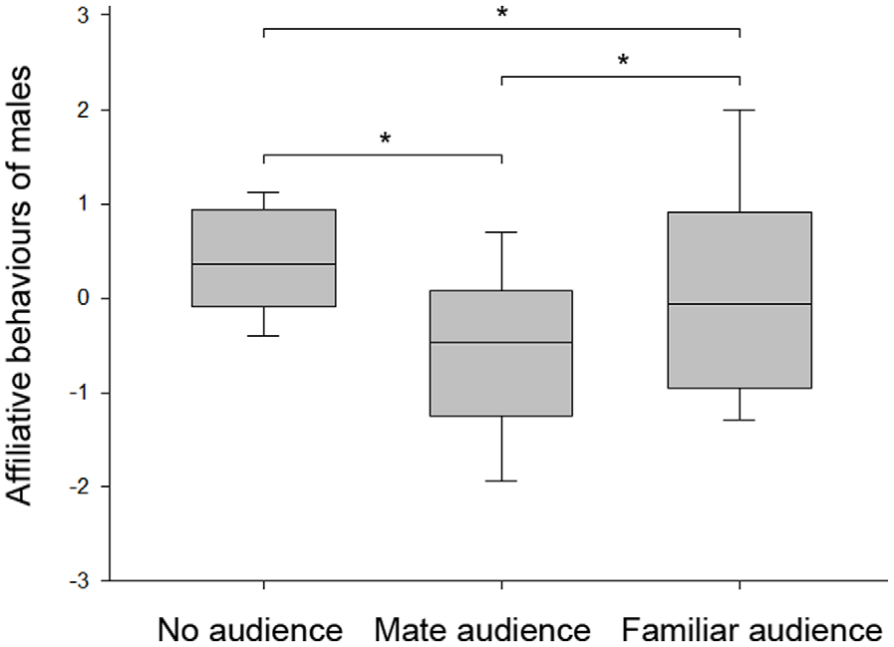
Experiment 1. Affiliative behaviours expressed by males according to the presented audience. Median, lower and upper quartiles are given. Error bars represent the 10^th^ and 90^th^ percentiles. *p<0.05. Statistical comparison: one way repeated measures ANOVA followed by Student-Newman-Keuls post hoc tests.

During an extra-pair interaction with a sexually receptive female, males significantly adjusted their affiliative behaviours to the behaviours of the interacting female by courting more if she was affiliative (LMM n°1: adjusted R^2^ = 0.27; F_(20,20)_ = 5.96; *p* = 0.019) and by courting less if she was aggressive (LMM n°1: adjusted R^2^ = −0.23; F_(20,20)_ = 5.40; *p* = 0.025) (Table 1). The behaviour of interacting females did not significantly change between experimental conditions (one way repeated measures ANOVA: F_(2,20)_ = 3.21; *p* = 0.2 for affiliative behaviours and F_(2,20)_ = 0.26; *p* = 0.87 for agonistic behaviours). More strikingly, males significantly adjusted their courting behaviour toward the interacting female according to the different audiences (one way repeated measures ANOVA: F_(2,20)_ = 10.77; *p* = 0.005). They significantly courted more: (i) without an audience than in the presence of a familiar audience (SNK: q = 4.03; *p*<0.05, N = 21), (ii) without an audience than in the presence of the mate (SNK: q = 4.61; *p*<0.05, N = 21 ) and (iii) in the presence of a familiar female than in the presence of the mate (SNK: q = 2.86; *p*<0.05; N = 21) (Fig. 2). To the contrary of courting behaviours, the analysis we performed on males' agonistic behaviours was not significant (LMM n°2: *p* = 0.2) (Table 1).

**Table 1:** Experiment 1: synthesis of the LMM analysis performed on the affiliative behaviours of males.

Model	Dependant variable	Explanatory variables	Adjusted R^2^	*F_(20, 20)_*	*p*
LMM n°1	Affiliative behaviours of ♂	Experimental condition	NA*	7.80	0.001
		Affiliative behaviours of interacting ♀	0.27	5.96	0.019
		Agonistic behaviours of interacting ♀	−0.23	5.40	0.025
LMM n°2	Agonistic behaviours of ♂	Experimental condition	NA*	2.49	0.09

* NA: not available.

#### Discussion

This experiment demonstrates that male canaries adjust their extra-pair behaviour to the presence of a social audience, as one could have expected from previous studies [12], [13]. Indeed, males courted less in the presence than in the absence of a female in audience and this result could not be explained by the behaviours of the interacting females or by those of the audiences as none of them behaved differently between experimental conditions. This result confirms the existence of audience effects during an extra-pair interaction as seen in the budgerigars *Melopsittacus undulatus* [30]. It is not surprising as both canaries and budgerigars are socially monogamous [30], [33] and in both species, males are an important resource for females as they feed them during the incubation period [39]. The results could be different if similar experiments were conducted in polygynous species or in species where paternal cares do not exist: because of different selection pressures, males could either court more in the presence of an audience (e.g. if females express mate choice copying [40]) or could express no audience effects.

More interestingly, these results show that male domestic canaries can adjust their behaviour according to the social bond they share with the audience. Indeed, subjects courted less in the presence of their mate than in the presence of a familiar female. This suggests that males suffer costs while engaging in extra-pair behaviours in the presence of their mate. For further studies, it could be of interest to study if this situation could cause females to decrease their reproductive investment [41], seek more extra-pair opportunities [15] or ‘divorce’ [42]. Similarly, males courted less in the presence of a familiar female than without an audience. One hypothesis to explain this result is that males might face a trade-off for their time, their attention and/or their motivation between the assessment of the female in audience and their affiliative behaviour with the interacting female.

At last, regardless of the experimental condition, males matched [24] their behaviours to those of the female they interacted with by courting more if she was affiliative (and to the contrary by courting less if she was aggressive).

### Experiment 2: effect of a mate/familiar audience on the competition behaviour of males

#### Subjects

In this experiment, we randomly assigned 20 males used in experiment 1 to create ten dyads.

#### Experimental design

The competition experiment was similar to the extra-pair experiment (Fig. 1) but: (i) a competitor male replaced the interacting female. (ii) Subjects were deprived for food for 2 hours before the experiment, an adequate duration to elicit competition for food [7]. Tests started with the experimenter placing seeds, mash and apple into the cage. (iii) According to the phase, the audience cage: (a) was empty, (b) contained a familiar female for the subject (the mate of the competitor male), or (c) the mate of the subject (a familiar female for competitor male).

#### Data analysis

Because male-male interactions do not include affiliative behaviours, we performed the same analysis as in experiment 1 but on males' agonistic behaviours only with LMM n°3 having: (i) the experimental condition and behaviours of the both the competitor male and female audiences as fixed effects and (ii) the identity of both the subjects and the dyads as random effects.

#### Results

The PCA we performed on males' behaviours had one main axis that explained 53.65% of the variance (eigenvalue: 2.20) and regrouped agonistic behaviours (eigenvectors: attack calls: 0.80; initiated threats: 0.80; initiated attacks: 0.90). This synthetic variable followed a Johnson's Si distribution (γ = −0.67; δ = 1.61; θ = −1.87; σ = 1) what allowed us normalizing it using Johnson's transforms [38] (Shapiro-Wilk test: W = 0.90; N = 20; *p* = 0.23).

**Figure 3:**
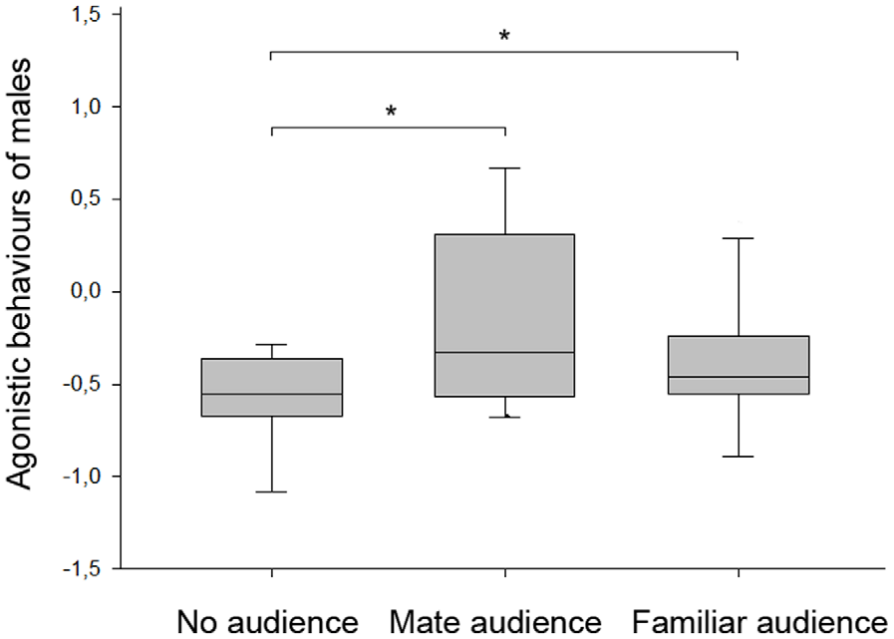
Experiment 2. Agonistic behaviours expressed by males according to the presented audience. Median, lower and upper quartiles are given. Error bars represent the 10^th^ and 90^th^ percentiles. *p<0.05. Statistical comparison: one way repeated measures ANOVA followed by Student-Newman-Keuls post hoc tests.

During a competition for food with a male competitor, males did not adjust their agonistic behaviour to the male competitor behaviour. This variable was removed during model selection. To the contrary, males adjusted their agonistic behaviour to the behaviour of females in audience by significantly attacking less the competitor male if she was affiliative (LMM n°3: adjusted R^2^ = −0.15; F_(19,19)_ = 7.03; *p* = 0.01) (Table 2). The behaviour of females in audience did not significantly change between experimental conditions (one way repeated measures ANOVA: F_(2,20)_ = 3.21; *p* = 0.2 for affiliative behaviours and F_(2,20)_ = 0.26; *p* = 0.87 for agonistic behaviours). In addition, males adjusted their agonistic behaviours toward the competitor male to the different audiences (one way repeated measures ANOVA: F_(2,19)_ = 7.52; *p* = 0.023). They were significantly more agressive: (i) in the presence of a familiar audience than without an audience (SNK test: q = 3.86; *p*<0.05; N = 20) and (ii) in the presence of the mate than without an audience (SNK test: q = 3.38; *p*<0.05; N = 20). But males did not behave differently in the presence of a familiar female than in the presence of the mate (SNK: q = 2.38; *p*>0.05; N = 20) (Fig. 3).

**Table 2:** Experiment 2: synthesis of the LMM analysis performed on the agonistic behaviours of males.

Model	Dependant variable	Explanatory variables	Adjusted R^2^	*F_(19, 19)_*	*p*
LMM n°3	Agonistic behaviours of ♂	Experimental condition	NA*	7.85	0.001
		Affiliative behaviours of ♀ audiences	−0.15	7.03	0.01

*NA: not available.

#### Discussion

This experiment demonstrates that male canaries behave differently according to the presence of the audience when they are engaged in a male-male competition for food. Indeed, males were more aggressive in the presence than in the absence of a female in audience. This audience effect is similar to those found in the guppy *Poecilia reticulata* [21] and the field cricket *Gryllus bimaculatus* [22] where males were also more aggressive in the presence of a female in audience. However, our results are surprising because previous eavesdropping experiments showed that female domestic canaries preferred the losers of physical interactions [13]; one could have expected that males become less aggressive in the presence of a female. Two hypotheses can be formulated to explain this discrepancy. First, males were paired in the present experiment and it could be possible that audience effects on male-male interaction could vary according to the paring status of these males; one can not exclude that unpaired males could have been less aggressive in the presence of a female. Second, and more likely, it can be explained by the experimental design used in Amy et al. [13]: females monitored a food contest between males and were allowed to choose between the two observed males right after the competition. Therefore, the authors might have observed a social avoidance of irritated males by females rather than a sexual preference. Indeed, approaching an aggressive individual right after a fight might be risky for a female.

Interestingly, contrary to the extra-pair experiment, males did not adjust their behaviour to the social bond they share with the audience: there were no differences in males' aggressiveness in the presence of the mate and in the presence of a familiar female. This absence of difference could be explained if the presence of these different audiences (mate of familiar female) has similar costs for males. One could assume that males losing a contest would suffer a decrease in their reproductive success in both situations: eavesdropping familiar females would not choose them as sexual partners (e.g. [9]) while eavesdropping mates would engage more in extra-pair copulations (e.g. [14]).

At last, regardless of the experimental condition, males matched [24] their behaviours to those of the females in audience: the more affiliative the female in audience was, the less aggressive males were. This result can be explained by a trade-off that males might face for their time, their attention and/or their motivation between the assessment of the female in audience and the interaction with the competitor male.

## Discussion

The method used in this study (repeated measures and model selection) allowed to disentangle the audience effects from behaviour matching [24] as it allows to separate the influence of the individual who interacts with the subject from the audience effect.

Our results provide new insights on sociality in canaries as, to our knowledge, audience effects had never been studied in this species.

More interestingly, these two experiments reveal astonishing behavioural plasticity as males adapted their behaviours: (i) to the context (they shifted the behaviours concerned by audience effect from affiliative to agonistic), (ii) to the social bonds they share with the audience (only during the the extra-pair interaction) and (iii) to the behaviours of both the audiences and the individual who interacts with them. Such results raise the question of the extent to which animals can be aware of social constraints and adapt their behaviour accordingly, especially if we put our results in relation with works that showed that animals also pay attention to the social relationships between the audiences [28] and to their hierarchical status [27]. This stresses the importance to study the cognitive processes involved in this regulation of social behaviours (e.g. [43]) especially considering that canaries, contrary to parrots, blue jays or ravens, are known neither for having high cognitive abilities nor for having a complex social life that could explain the evolution of this finely mediated audience effect.

At last, this study also raises the question of the reliability of the information obtained by bystanders and eavesdroppers: could perverted information impact the sexual preferences expressed by females and, if so, would females adopt specific strategies to gain access to reliable indicators of the quality of males [44]?
